# Parameter-Free Multiscale Simulation Realising Quantitative Prediction of Hole and Electron Mobilities in Organic Amorphous System with Multiple Frontier Orbitals

**DOI:** 10.1038/s41598-018-31722-w

**Published:** 2018-09-07

**Authors:** Shosei Kubo, Hironori Kaji

**Affiliations:** Institute for Chemical Research, Kyoto University, Uji, Kyoto, 611-0011 Japan

## Abstract

In amorphous organic semiconducting systems, hole and electron transfer has been considered to occur based on the overlap of highest occupied molecular orbitals (HOMOs) and that of lowest unoccupied molecular orbitals (LUMOs) between two adjacent molecules, respectively. Other molecular orbitals (MOs), HOMO−1, HOMO−2, … and LUMO+1, LUMO+2, …, have been neglected in charge transport calculations. However, these MOs could potentially contribute to charge transport. In this study, our multiscale simulations show that carriers are effectively transported not only via HOMOs or LUMOs but also via other MOs when the MOs are close in energy. Because these multiple MOs are active in charge transports, here we call them *multiple frontier orbitals*. Molecules with multiple frontier orbitals are found to possess high carrier mobility. The findings in this study provide guidelines to aid design of materials with excellent charge transport properties.

## Introduction

The charge transport processes in organic semiconductors are important to understand the performance of organic semiconductor devices such as organic light-emitting diodes (OLEDs)^1^, organic field-effect transistors^2^, and organic photovoltaics^3^. Much effort has been devoted to investigating the charge transport properties of organic semiconductors, typically in terms of charge mobility *μ*^4^. In organic “*amorphous*” systems, one successful description of charge transport is called the Gaussian disorder model (GDM)^5^. However, because this model, which is based on hopping-type charge transport, does not take actual molecules into account, it is not straightforward to unveil the relationship between the molecular structure and charge transport properties of a material, even though this relationship is important for material designs and device optimisation. An explicit consideration of molecules in aggregates should be used in charge transport models to access molecular-level insight into charge transport processes^6–16^.

In our previous studies, a hopping-type charge transport process in amorphous aggregates of materials used in OLEDs was investigated by a multiscale simulation including quantum chemical, molecular dynamics (MD), and kinetic Monte Carlo (kMC) calculations^12,15^. To simulate charge transport in organic amorphous systems, an amorphous aggregate is generated by an MD simulation. In the hopping-type charge transport model, the elementary step of charge transport is charge hopping between two adjacent molecules of relevance. For various molecules or molecular pairs in the MD-constructed amorphous aggregate, the hopping site energy difference, electronic coupling, and reorganisation energy associated with charge transfer are calculated, which are crucial factors determining the rate of charge hopping^4^. Starting from the molecular structure of an organic material, multiscale simulation enables us to hierarchically link the microscopic (structural and/or electronic) details to the macroscopic charge transport properties.

Disorder in an amorphous system yields a *distribution* of hopping site energy and *distribution* of electronic coupling between adjacent hopping sites^4,11^. By explicitly considering these distributions, our multiscale simulations reasonably reproduced an experimentally observed positive dependence of *μ* versus electric field (*F*), ${\rm{\partial }}\,\mathrm{ln}\,\mu /{\rm{\partial }}{F}^{1/2} > 0$, which is called Poole–Frenkel-type field dependence. The experimental hole mobility was well reproduced by the multiscale simulation *without use of adjustable parameters*^15^. However, for electron transport, the agreement between simulated and experimental mobilities was not satisfactory. Because accurate physical insight into charge transport phenomena in actual organic devices has not been fully unravelled, the molecular- to device-level (i.e., multiscale) approach to investigate charge transport still needs to be further refined.

**Figure 1:**
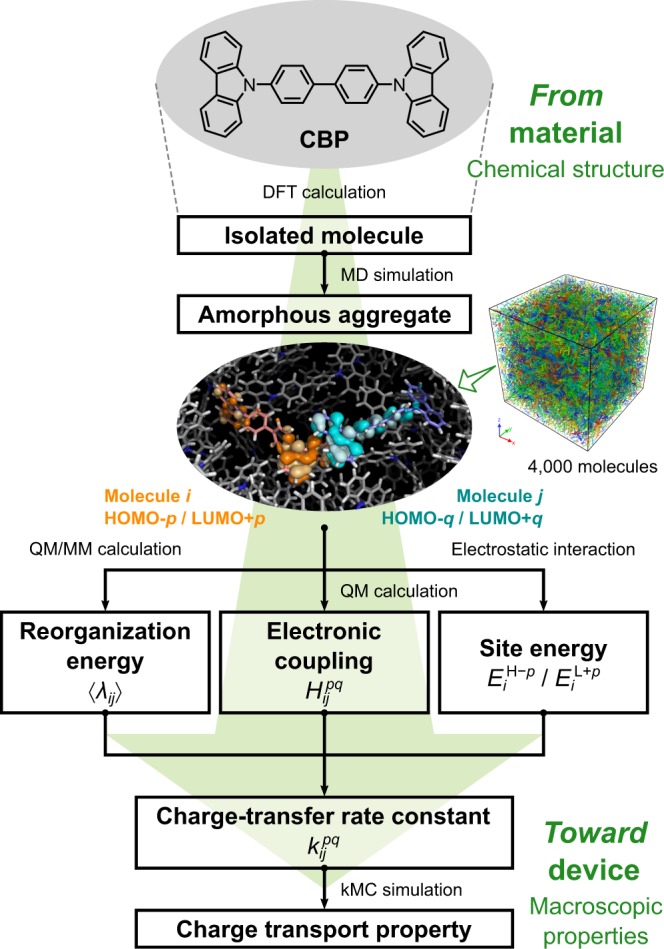
Molecular structure of CBP and multiscale simulation flowchart of the charge transport process.

It is currently unclear if charge transport occurs only through the highest occupied molecular orbital (HOMO) or lowest unoccupied molecular orbital (LUMO) of a molecule. We envisaged that not only the HOMO or LUMO but also other molecular orbitals (MOs) may potentially contribute to charge transport. However, all previous studies simulating charge transport in organic “*amorphous*” systems do not consider MOs other than the HOMO and LUMO, except for crystalline^17,18^ or liquid crystalline^19,20^ systems. In this study, we consider the involvement of multiple MOs in charge transport in organic “*amorphous*” systems. Figure 1 shows a flowchart of our new multiscale charge transport simulation. This multiscale simulation results in excellent agreement of calculated and experimental mobilities for both hole and electron transport, as shown later. The simulations clearly show that not only the HOMO and LUMO but also other MOs are *active* in charge transport. From this viewpoint, we call such MOs *multiple frontier orbitals*, which are analogous to frontier orbitals in the context of chemical reactions. This study also shows that these other MOs, such as lower-lying occupied MOs (HOMO−1, HOMO−2, …) or higher-lying unoccupied MOs (LUMO+1, LUMO+2, …), increase charge mobility when the energy levels lie close to the HOMO or LUMO (i.e., are degenerate or quasi-degenerate). Thus, molecules with (quasi-)degenerate MOs that can behave as active multiple frontier orbitals are promising to realise highly efficient charge carrier transport.

**Figure 2:**
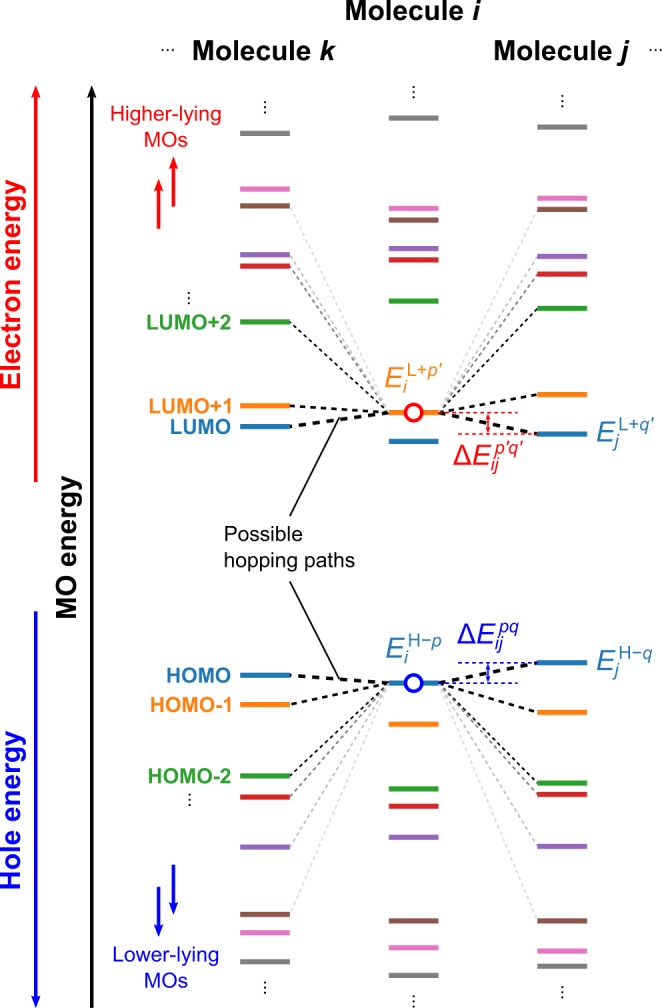
Schematic illustration of the energy landscape of site energies including lower- and higher-lying MOs as well as the HOMO and LUMO. ${E}_{i}^{{\rm{H}}-p}$ and ${E}_{i}^{{\rm{L}}+{p}^{^{\prime} }}$ denote the site energies of the HOMO−*p* and LUMO+*p*′ of the *i*th molecule, and ${E}_{j}^{{\rm{H}}-q}$ and ${E}_{j}^{{\rm{L}}+{q}^{^{\prime} }}$ are those of the HOMO−*q* and LUMO+*q*′ of the *j*th molecule. Dashed lines connecting two MOs represent possible hopping paths for a carrier on the HOMO−*p* or LUMO+*p*′ of the *i*th molecule (represented as red and blue open circles, respectively) to various MOs of adjacent molecules *j*, *k*, etc. The thickness of the black dashed lines reflects the hopping rate constant.

Here, we denote HOMO−*p* and LUMO+*p* as the *p*th MO (*p* = 0, 1, …). Note that HOMO−0 and LUMO+0 (i.e. *p* = 0) indicate the HOMO and LUMO, respectively. Molecules in an amorphous aggregate have different conformations, resulting in different hopping site energies. We investigated the effect of conformation on site energy, which has not been considered in our previous works^12,15^. In addition, we included intermolecular effects, an electrostatic interaction and polarisation effect of neighbouring molecules in an amorphous aggregate, in the calculation of site energy as in ref.^15^. The site energy was calculated including these effects based on the energy of the corresponding MO for an isolated molecule. Such intra- and intermolecular effects cause a distribution of site energy, which is called *energetic disorder* or *diagonal disorder*. We defined the site energies for the HOMO−*p* and LUMO+*p* of the *i*th molecule in the amorphous aggregate as

1 $${E}_{i}^{{\rm{H}}-p}={\rm{\Delta }}{\epsilon }_{i}-{\epsilon }_{i}^{{\rm{H}}-p}\,\text{and}\,{E}_{i}^{{\rm{L}}+p}={\rm{\Delta }}{\epsilon }_{i}+{\epsilon }_{i}^{{\rm{L}}+p}$$

where the superscript H−*p* and L+*p* indicate the HOMO−*p* and LUMO+*p*, respectively. ${\rm{\Delta }}{\epsilon }_{i}$ is the sum of the electrostatic interaction and polarisation effect of the carrier on the *i*th molecule. ${\epsilon }_{i}^{{\rm{H}}-p}$ and ${\epsilon }_{i}^{{\rm{L}}+p}$ are the eigenvalues of the HOMO−*p* and LUMO+*p*, respectively, of the *i*th molecule in an isolated state retaining its molecular structure in the amorphous aggregate; these eigenvalues include the conformational effect of each molecule. Different site energies for respective molecules result in a rugged energy landscape (Fig. 2). A charge in a certain MO can hop to an adjacent MO. The calculations in this study were conducted for multiple adjacent molecular sites with numerous different MOs for each molecule.

## Results

**Figure 3:**
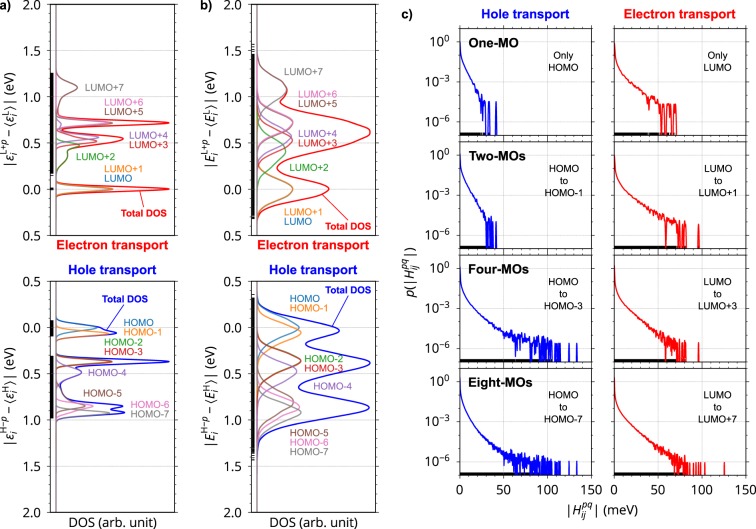
(**a**,**b**) Density of states (DOS) for the amorphous CBP system calculated for (**a**) the site energy with intramolecular effect (without considering intermolecular interactions), ${\epsilon }_{i}^{{\rm{H}}-p}$ or ${\epsilon }_{i}^{{\rm{L}}+p}$, which corresponds to MO energy, and (**b**) the site energy with both intra- and intermolecular interactions, ${E}_{i}^{{\rm{H}}-p}={\rm{\Delta }}{\epsilon }_{i}-{\epsilon }_{i}^{{\rm{H}}-p}$ or ${E}_{i}^{{\rm{L}}+p}={\rm{\Delta }}{\epsilon }_{i}+{\epsilon }_{i}^{{\rm{L}}+p}$. A Gaussian broadening of 25 meV was applied. The PDOS for HOMO to HOMO−7 and LUMO to LUMO+7 are also shown. Total DOS is plotted as a superposition of the PDOS for the eight-MOs. ${\epsilon }_{i}^{{\rm{H}}}$, ${\epsilon }_{i}^{{\rm{L}}}$, ${E}_{i}^{{\rm{H}}}$, and ${E}_{i}^{{\rm{L}}}$ denote the peak energies of the PDOS for HOMO or LUMO of the corresponding cases. The energies are represented as relative values to the respective peak energies, ${\epsilon }_{i}^{{\rm{H}}}$, ${\epsilon }_{i}^{{\rm{L}}}$, ${E}_{i}^{{\rm{H}}}$, and ${E}_{i}^{{\rm{L}}}$. (**c**) Probability densities of electronic coupling $|{H}_{ij}^{pq}|$, $p(|{H}_{ij}^{pq}|)$, for the cases in which one-, two-, four-, and eight-MOs were included.

Figure 3a,b show the density of states (DOS) for an MD-constructed amorphous aggregate composed of 4,000 molecules of 4,4′-bis(*N*-carbazolyl)−1,1′-biphenyl (CBP; its molecular structure is shown in Fig. 1). The total DOS calculated for ${\epsilon }_{i}^{{\rm{H}}-p}$ or ${\epsilon }_{i}^{{\rm{L}}+p}$, which reflects the effect of various conformations of molecules in the amorphous aggregate, is distributed, even though the total DOS does not contain the effect of intermolecular interactions (Fig. 3a). This clearly indicates that the energetic disorder partly originates from ${\epsilon }_{i}^{{\rm{H}}-p}$ or ${\epsilon }_{i}^{{\rm{L}}+p}$; that is, different molecular conformations. When both ${\rm{\Delta }}{\epsilon }_{i}$ (intermolecular effect) and ${\epsilon }_{i}^{{\rm{H}}-p}$ or ${\epsilon }_{i}^{{\rm{L}}+p}$ (intramolecular effect) were considered in the site energy calculations [equation (1)], the total DOS turned to that in Fig. 3b, which was much broader than that considering only ${\epsilon }_{i}^{{\rm{H}}-p}$ or ${\epsilon }_{i}^{{\rm{L}}+p}$ (Fig. 3a). Here, we call the DOS for respective MOs partial DOS (PDOS). According to the GDM, the most well-known phenomenological charge transport model, a Gaussian distribution is postulated for HOMO and LUMO^5^. In contrast, mostly Gaussian shaped PDOS was obtained for all the MOs without any assumption in our model. The standard deviation was calculated to be 106 meV for HOMO, which is in good agreement with that determined by thin film transistor technique (95 meV) in terms of the GDM^21^ (no reports are found for electron transport). It is noteworthy that the PDOS for the HOMO−1 and LUMO+1 mostly overlapped with those for the HOMO and LUMO, respectively (Fig. 3b); the average energy level difference between the HOMO and HOMO−1 was 55.1 meV and that between the LUMO and LUMO+1 was 3.4 meV. These results suggest that both the HOMO−1 and LUMO+1 will contribute to charge transport, which is actually the case, as shown later. The energy levels of HOMO−*p* and LUMO+*p* with $p\ge 2$ are energetically separated from those with $p\le 1$ (larger than 0.3 eV on average), suggesting these MOs make a smaller or negligible contribution to charge transport.

Next, we consider *structural disorder* (known also as *off-diagonal disorder*); that is, the distribution of electronic coupling. Here, the electronic coupling between the HOMO−*p* or LUMO+*p* of the *i*th molecule and the HOMO−*q* or LUMO+*q* of the *j*th molecule is denoted as ${H}_{ij}^{pq}$ (*i*, *j* = 1, …, 4000; *p*, *q* = 0, 1, …). Figure 3c compares the probability densities of $|{H}_{ij}^{pq}|$, $p(|{H}_{ij}^{pq}|)$, in the cases in which one-, two-, four-, and eight-MOs were considered. When multiple MOs were included, the distribution of $|{H}_{ij}^{pq}|$ was wider than that for the one-MO. The maximum values of $|{H}_{ij}^{pq}|$ in the eight-MO model, 133 and 125 meV for holes and electrons, respectively, are larger than those in the one-MO model of 41 and 70 meV, respectively. This suggests that charges will hop effectively using MOs with $p\ge 1$ in terms of $|{H}_{ij}^{pq}|$. However, the following kMC calculations clearly reveal that the MOs with $p\ge 2$ do not contribute to charge transport because of their unfavourable energy levels (Fig. 3b).

**Figure 4:**
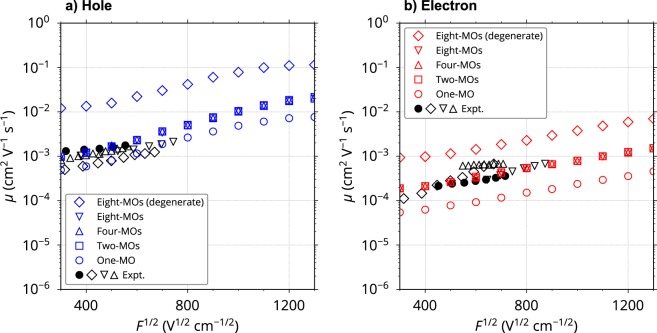
Calculated and experimental charge mobilities *μ* for (**a**) hole and (**b**) electron transport as a function of *F*^1/2^. The calculated results for two-, four-, and eight-MO models overlapped. The coloured open diamonds represent the mobilities assuming that all eight-MOs are quasi-degenerate; they possess an energy difference which originates only from the intermolecular interaction (i.e., ${E}_{i}^{{\rm{H}}-p}={\rm{\Delta }}{\epsilon }_{i}$ and ${E}_{i}^{{\rm{L}}-p}={\rm{\Delta }}{\epsilon }_{i}$; its standard deviation is about 0.1 eV). Experimental data (black symbols) from ref.^15^ (filled circles), ref.^10^ (open diamonds), ref.^23^ (open downward triangles), and ref.^24^ (open upward triangles) are also shown.

The rate constant of charge hopping from the *p*th MO of the *i*th molecule to the *q*th MO of the *j*th molecule, ${k}_{ij}^{pq}$, was calculated based on Marcus theory^22^. Using ${k}_{ij}^{pq}$, we performed charge transport kMC simulations for a thin film consisting of the MD-constructed amorphous aggregate. Computational details are provided in Methods section. Figure 4 shows the calculated hole and electron mobilities as a function of *F*^1/2^ considering one-, two-, four-, and eight-MOs as hopping sites. The calculated mobilities considering two-MOs (HOMO−1 or LUMO+1 in addition to the HOMO or LUMO) are 2–3 times higher than those considering only one-MO (HOMO or LUMO). The results of the case of one-MO almost agreed with those of our previous model^15^. Much lower- and higher-lying MOs (HOMO−2, HOMO−3, …, and LUMO+2, LUMO+3, …) did not influence mobilities as was expected from Fig. 3c. This result indicates that the MOs contribute to charge transport only when they lie close in energy, even if the relevant electronic coupling is considerable. Experimental data obtained by a time-of-flight (TOF) method^10,15,23,24^ are also shown in Fig. 4. The two-MO model resulted in excellent agreement with the experimental data for both hole and electron transport. This is a significant improvement from our previous model where the agreement for electron mobility was poor due to the ignorance of the contribution from LUMO+1. Of course, the four- and eight-MO models also reproduced the experimental data, but these models are less computationally efficient than the two-MO model.

**Figure 5:**
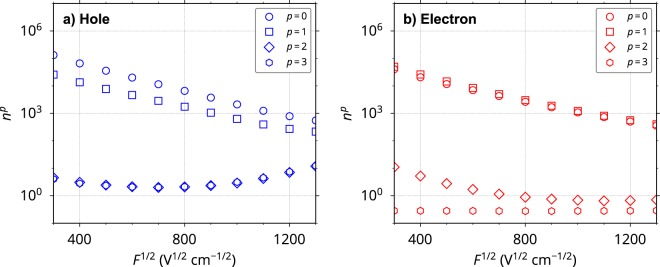
Number of incoming carriers from all adjacent MOs to *p*th MOs in the amorphous system, *n*^*p*^, as a function of *F*^1/2^ for (**a**) hole and (**b**) electron transport in the four-MO model. *n*^*p*^ is averaged over 30,000 trials of the kMC simulation.

To obtain further details, we calculated the number of carrier hops from the *q*th MO of the *j*th molecule to the *p*th MO of the *i*th molecule, ${n}_{ji}^{qp}$ (note that ${n}_{ij}^{pq}$ represents the hopping in the opposite direction). Using ${n}_{ji}^{qp}$, the number of incoming carriers from all adjacent MOs to the *p*th MO of the *i*th molecule can be expressed as ${n}_{i}^{p}=\sum _{j,q}{n}_{ji}^{qp}$. Figure 5 shows the sum of ${n}_{i}^{p}$ over all 4,000 molecules in the aggregate, ${n}^{p}=\sum _{i}{n}_{i}^{p}$, which reflects the frequency of using the *p*th MOs in the amorphous system during charge transport over a distance of 100 nm along ***F*** (all values are the average of 30,000 trials of the kMC simulation). It is clear that the HOMO−1 and LUMO+1 were frequently used as well as the HOMO and LUMO during hole and electron transport, respectively. In electron transport, the contributions of the LUMO+1 and LUMO are nearly equal (*n*^1^ ≈ *n*^0^). In contrast, the HOMO−1 was used slightly less frequently than the HOMO for hole transport (${n}^{1} < {n}^{0}$), reflecting the smaller energy difference between the LUMO and LUMO+1 (3.4 meV on average) compared to that between the HOMO and HOMO−1 (55.1 meV on average). This readily explains the larger increase of electron mobility than that of hole mobility when we include the contributions of the HOMO−1 and LUMO+1 in addition to the HOMO and LUMO (Fig. 4). Figure 5 also reveals that the contributions of MOs with $p\ge 2$ were much smaller than those of the HOMO, HOMO−1, LUMO, and LUMO+1 (see Supplementary Fig. S3 for MOs with $p\ge 4$). This is consistent with the negligible contributions of these MOs to the charge mobilities in Fig. 4. According to Marcus equation [equation (3) in Methods section], the rate constant for charge hopping from lower-lying MO (HOMO−*p* with large *p*) to HOMO is larger than that for hopping between two HOMOs due to the large difference in site energy. Therefore, holes occupying HOMO−*p* with large *p* were immediately transferred to HOMO or HOMO−1 during a few tens of hopping steps in our simulations, which were negligibly short compared to the whole charge hopping process (shorter than 1/100 of the whole process, see Supplementary Fig. S4a), in spite that no assumption was made for a probability of charge injection among the multiple MOs and that the internal conversion of carriers was not considered. In addition to HOMO and HOMO−1, HOMO−2 and HOMO−3 were used sometimes (Supplementary Fig. S4a), because the high energy edges of HOMO−2 and HOMO−3 are within the energy distribution of HOMO and HOMO−1 (see Fig. 3b). This indicates that the phenomenon originates from the distribution of site energy, that is, energetic disorder. The same picture holds true for electron hopping (Supplementary Fig. S4b and Fig. 3b).

The above discussion and Fig. 4 reveal that MOs with close energy levels to those of HOMO and LUMO are involved in charge transport in organic amorphous systems. These MOs are *active* in charge transport, so we call such multiple MOs “*multiple frontier orbitals*”. The above results and discussion also indicate that high charge mobility can be achieved when as many as possible MOs lie close in energy to the HOMO or LUMO (i.e., are degenerate or quasi-degenerate). We obtained very high hole and electron mobilities (blue and red open diamonds in Fig. 4a,b, respectively) when the eight-MOs in our amorphous system were close in energy. The hole and electron mobilities of the eight-MO model with degenerate MOs were more than one order of magnitude higher than those of the one-MO model without degenerate MOs (blue and red open circles in Fig. 4a,b, respectively). We found that all the MOs were used almost equally when the eight-MOs were close in energy, as shown in Supplementary Fig. S5 (*n*^*p*^ versus *F*^1/2^).

**Figure 6:**
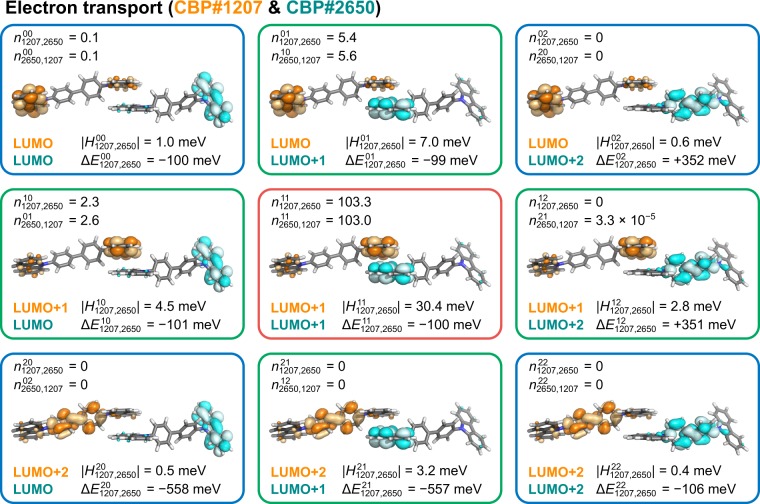
Examples of MO pairs for electron transport with $p\le 2$ (LUMO to LUMO+2), depicted with an isosurface of 0.02 a.u. The red, green, and blue boxes indicate the MO pairs in which the two MOs exhibit sufficient overlap (red), one of the relevant MOs is localised on a contacting part of the relevant molecule (green), and both of the relevant MOs are localised far from the contacting part (blue), respectively. The electronic coupling $|{H}_{ij}^{pq}|$, site energy difference ${\rm{\Delta }}{E}_{ij}^{pq}$, and number of hops between the *p*th MO of the *i*th molecule and the *q*th MO of the *j*th molecule, ${n}_{ij}^{pq}$ and ${n}_{ji}^{qp}$, for each pair are also provided. ${n}_{ij}^{pq}$ and ${n}_{ji}^{qp}$ are averaged over 30,000 trials of the kMC simulation at *F*^1/2^ = 800 V^1/2^ cm^−1/2^.

Here, we present the detailed molecular-level analysis of the contributions of nine MO pairs that are combinations of three MOs, LUMO, LUMO+1, and LUMO+2 for electron transport (the results for hole transport are summarised in Supplementary Fig. S6). Figure 6 shows the spatial distributions of LUMO to LUMO+2 for the pair consisting of the 1207th and 2650th molecules in our amorphous system as an example. This is one of the most frequently used molecular pairs in electron transport. It is worth to note that the LUMO are localised at one of the two carbazole moieties and the LUMO+1 are localised at the opposite carbazole unit, resulting in the quasi-degenerate MOs as discussed above. Figure 6 also shows the number of electron hops between the LUMO+*p* of the 1207th molecule and the LUMO+*q* of the 2650th molecule, ${n}_{1207,2650}^{pq}$ and ${n}_{2650,1257}^{qp}$. There are various intermolecular packings in the amorphous aggregate, and the intermolecular orientations and distances between two molecules strongly affect their electronic coupling. In Fig. 6, although the intermolecular orientation and distance remain the same, the intermolecular overlap between two MOs strongly depends on the spatial distribution of the respective MOs. The various overlaps influence the electronic coupling. The electronic coupling between the two LUMO+1’s of the two molecules ($|{H}_{1207,2650}^{11}|$ = 30.4 meV; the MO pair in a red box in Fig. 6) is the largest among the nine MO pairs because of the sufficient overlap between the two MOs (both MOs are localised at the contacting part). In contrast, the electronic couplings are smaller when one (MO pairs in green boxes in Fig. 6) or both (MO pairs in blue boxes) of the relevant MOs are localised far from the contacting part of the two molecules. Figure 6 also shows the site energy difference, ${\rm{\Delta }}{E}_{1207,2650}^{pq}$ where $p,q\le 2$. We clearly found that the LUMO+2 was not used for electron transport because the site energy differences between the LUMO and LUMO+2 or the LUMO+1 and LUMO+2 were larger than 0.3 eV, and the values of ${n}_{1207,2650}^{pq}$ were negligibly small; that is, it is energetically unfavourable for the charge to hop to the LUMO+2 for these MO pairs. The site energy differences between the two LUMOs, ${\rm{\Delta }}{E}_{1207,2650}^{00}$, between the LUMO and LUMO+1, ${\rm{\Delta }}{E}_{1207,2650}^{01}$ and ${\rm{\Delta }}{E}_{1207,2650}^{10}$, and between the two LUMO+1’s, ${\rm{\Delta }}{E}_{1207,2650}^{11}$, were comparable (ca. 100 meV). Thus, the number of hops for these MO pairs is solely determined by the electronic coupling between the two relevant MOs; the largest electronic coupling, which is found for the two LUMO+1’s, results in the largest number of hops (${n}_{1207,2650}^{11}$ = 103.3 and ${n}_{2650,1207}^{11}$ = 103.0).

The same discussion holds for the whole amorphous system. Tables 1 and 2 show the number of hops from *p*th to *q*th MOs, ${n}^{pq}=\sum _{i,j}{n}_{ij}^{pq}$, for the whole system. It is found that the charge hopping involving not only the HOMO and LUMO but also the HOMO−1 and LUMO+1 strongly contributes to charge transport in the whole system. Tables 1 and 2 also clearly indicate that the MO pairs with $p\ge 2$ or $q\ge 2$ were rarely used during both hole and electron transport. The charge transport simulation including the charge hopping involving the HOMO−1 and LUMO+1 in addition to the HOMO and LUMO successfully described the charge transport properties for the amorphous aggregate of CBP.

**Table 1:** Number of carrier hops from *p*th to *q*th MOs summed over all possible molecular pairs, ${n}^{pq}=\sum _{i,j}{n}_{ij}^{pq}$, for hole transport. All values are averaged over 30,000 trials of the kMC simulation at *F*^1/2^ = 800 V^1/2^ cm^−1/2^.

*n*^*pq*^ (hole)	HOMO	HOMO−1	HOMO−2	HOMO−3
HOMO	5118.2	1454.6	0.2	0.3
HOMO−1	1454.8	288.2	0.3	0.2
HOMO−2	0.3	0.4	0.6	0.7
HOMO−3	0.4	0.4	0.7	0.7

**Table 2:** Number of carrier hops from *p*th to *q*th MOs summed over all possible molecular pairs, ${n}^{pq}=\sum _{i,j}{n}_{ij}^{pq}$, for electron transport. All values are averaged over 30,000 trials of the kMC simulation at *F*^1/2^ = 800 V^1/2^ cm^−1/2^.

*n*^*pq*^ (electron)	LUMO	LUMO+1	LUMO+2	LUMO+3
LUMO	1030.6	1600.0	0.3	0.0006
LUMO+1	1600.0	1423.4	0.3	0.001
LUMO+2	0.4	0.4	0.03	0.008
LUMO+3	0.1	0.1	0.05	0.02

In conclusion, we proposed a multiscale simulation scheme that considers lower- and higher-lying MOs in addition to the HOMO and LUMO to investigate the charge transport process in amorphous systems of organic materials. To our knowledge, this is the first study that explicitly includes multiple MOs in the calculation of charge transport in an “*amorphous*” system. As for crystalline or liquid crystalline systems, a few reports considering lower- or higher-lying MOs were found^17–20^. In these studies, the electronic state of hopping sites was obtained using mathematical techniques, such as a symmetric orthogonalisation^17^ or symmetry-adapted linear combination^19,20^. However, these approaches have high computational cost or cannot be applied to systems without structural symmetry. In contrast, our simple simulation scheme can be widely applied irrespective of the structural symmetry of the system with reasonable cost. Our simulations quantitatively reproduced experimentally determined mobilities for both hole and electron transport. The detailed analysis revealed that not only the HOMO and LUMO but also the HOMO−*p* and LUMO+*p*′ contributed markedly to the charge transport in an amorphous solid when their energy levels were comparable to those of the HOMO or LUMO. Thus, multiple frontier orbitals can be actively involved in charge transport. These findings indicate that (quasi-)degenerate MOs play a crucial role in charge transport. Designing materials with multiple frontier orbitals will be a promising approach to realise excellent charge transport properties.

## Methods

### Quantum chemical calculations and MD simulations

Quantum chemical calculations and MD simulations were performed using the Gaussian 09 program package^25^ and LAMMPS program package^26^, respectively. We first performed a structure optimisation for an isolated CBP based on a density functional theory (DFT) calculation using the B3LYP functional^27,28^ and 6–31G* basis set. We note that the B3LYP functional was used only for the structure optimisation and the calculation of reorganisation energy (see below for details). The DFT-optimised CBP molecule was used as an initial single molecular structure to construct amorphous aggregates. To confirm the reproducibility of charge transport simulations, we generated two amorphous models consisting of 4,000 CBP molecules with different initial intermolecular structures by MD simulations. These structures are referred to as CBP-4000-A and CBP-4000-B. In the MD simulations, the Dreiding force field^29^ was used, and the bond lengths and angles of the DFT-optimised molecule were applied as the equilibrium bond length and angle parameters. The coulombic interaction between atoms was calculated based on the particle–particle-particle–mesh method^30^ using atomic partial charges for the DFT-optimised molecule determined by the Merz–Singh–Kollman scheme^31^. The MD simulations were performed as follows: (i) an NVT ensemble at 573 K for 0.1 ns, and (ii) an NPT ensemble at 298 K and 1.0 × 10^−4^ Pa for 1.0 ns using a Nosé–Hoover thermostat and barostat^32,33^. Supplementary Fig. S1 confirms that the density of the amorphous aggregates has reached equilibrium within the above MD simulation time. In more precise, site energy and electronic coupling fluctuate due to the molecular vibrations^34–37^. The calculation of site energy and electronic coupling over a long time interval provides reasonable statistical time averages, but is time-consuming. Here, we performed energy minimisation at the end of MD simulation. The resulting structures are with a side length and density of 14.6 nm and 1.04 g cm^−3^, respectively, for CBP-4000-A and 14.5 nm and 1.05 g cm^−3^, respectively, for CBP-4000-B. Unless otherwise noted, we used CBP-4000-A for the discussion in this study.

### MO energy and electronic coupling

The energy of the HOMO−*p* and LUMO+*p* of the *i*th molecule, ${\epsilon }_{i}^{{\rm{H}}-p}$ and ${\epsilon }_{i}^{{\rm{L}}+p}$, respectively, and the electronic coupling between the HOMO−*p* or LUMO+*p* of the *i*th molecule and the HOMO−*q* or LUMO+*q* of the *j*th molecule, ${H}_{ij}^{pq}$, were calculated based on the extended Hückel method^12,15,38,39^. ${H}_{ij}^{pq}$ is expressed as

2 $${H}_{ij}^{pq}=\frac{{\beta }_{ij}^{pq}-({\alpha }_{i}^{p}+{\alpha }_{j}^{q}){S}_{ij}^{pq}/2}{1-({S}_{ij}^{pq}{)}^{2}}$$

where ${\alpha }_{i}^{p}=\langle {\psi }_{i}^{p}|{\hat{H}}_{ij}|{\psi }_{i}^{p}\rangle$, ${\beta }_{ij}^{pq}=\langle {\psi }_{i}^{p}|{\hat{H}}_{ij}|{\psi }_{j}^{q}\rangle$, and ${S}_{ij}^{pq}=\langle {\psi }_{i}^{p}|{\psi }_{j}^{q}\rangle$. Here, ${\hat{H}}_{ij}$ is the Hamiltonian of *i*−*j* dimer system. ${\psi }_{i}^{p}$ and ${\psi }_{j}^{q}$ are the HOMO−*p* or LUMO+*p* of the *i*th molecule and the HOMO−*q* or LUMO+*q* of the *j*th molecule, respectively. To calculate ${H}_{ij}^{pq}$, the molecular pairs with a centre-to-centre distance of less than 2.5 nm (167,993 and 168,103 pairs for CBP-4000-A and CBP-4000-B, respectively) were included.

### Electrostatic and polarisation interactions of a carrier on the molecule

The calculation of the sum of the electrostatic interaction and polarisation effect of a carrier on the *i*th molecule, ${\rm{\Delta }}{\epsilon }_{i}$, is summarised in ref.^15^. The neighbouring molecules within a cutoff distance of 3.0 nm were considered in the calculation of ${\rm{\Delta }}{\epsilon }_{i}$. The AMOEBA polarisable force field^40^ was used to provide atomic polarisabilities to calculate the dipole moments induced on neighbouring molecules.

### Reorganisation energy

Reorganisation energies are commonly calculated for isolated molecules; however, it has been reported that intermolecular packing in an aggregated state influences reorganisation energy^15,41,42^. To incorporate the intermolecular steric influence of neighbouring molecules into the reorganisation energy, we used a quantum mechanics (QM)/molecular mechanics (MM) approach^43,44^. The reorganisation energy associated with the charge transfer from the *i*th to the *j*th molecule, *λ*_*ij*_, was calculated based on Nelsen’s four-point method^45^. DFT (B3LYP/6-31G*) and the Dreiding force field were used for the QM and MM regions, respectively. The neighbouring molecules within a cutoff distance of 3.0 nm were included in the MM region, and their geometries were frozen during the calculation. The atomic partial charges of the neighbouring molecules were embedded in the QM Hamiltonian. To lower the computational cost, we used an average value of *λ*_*ij*_, *λ*_*ij*_, over randomly sampled molecules [0.105 eV/0.373 eV (45 molecules in CBP-4000-A) and 0.112 eV/0.385 eV (50 molecules in CBP-4000-B) for hole/electron transport, respectively] in the calculation of charge-transfer rate constants.

### Charge transfer rate constant

Using the calculated values of $|{H}_{ij}^{pq}|$, ${E}_{i}^{{\rm{H}}-p}$ or ${E}_{i}^{{\rm{L}}+p}$, and *λ*_*ij*_, the rate constant of charge hopping from the *p*th MO of the *i*th molecule to the *q*th MO of the *j*th molecule, ${k}_{ij}^{pq}$, was calculated based on Marcus theory^22^:

3 $${k}_{ij}^{pq}=\frac{|{H}_{ij}^{pq}{|}^{2}}{\hslash }{(\frac{\pi }{\langle {\lambda }_{ij}\rangle {k}_{{\rm{B}}}T})}^{1/2}\exp [-\frac{(\langle {\lambda }_{ij}\rangle +{\rm{\Delta }}{E}_{ij}^{pq}-{q}_{{\rm{c}}}{\boldsymbol{F}}\cdot {{\boldsymbol{r}}}_{ij}{)}^{2}}{4\langle {\lambda }_{ij}\rangle {k}_{{\rm{B}}}T}]$$

where *ℏ* = *h*/(2π), *h* is the Planck constant, *k*_B_ is the Boltzmann constant, *q*_c_ is the charge of a carrier, ***F*** (|***F***| = *F*) is the external applied electric field, *T* is the absolute temperature, and ***r***_*ij*_ is the vector connecting the *i*th and *j*th molecules. Here, ${\rm{\Delta }}{E}_{ij}^{pq}$ is the difference between the site energies of the *p*th MO of the *i*th molecule and the *q*th MO of the *j*th molecule, which is defined as ${\rm{\Delta }}{E}_{ij}^{pq}={E}_{j}^{{\rm{H}}-q}-{E}_{i}^{{\rm{H}}-p}$ or ${\rm{\Delta }}{E}_{ij}^{pq}={E}_{j}^{{\rm{L}}+q}-{E}_{i}^{{\rm{L}}+p}$ for hole or electron transfer, respectively.

### Kinetic Monte Carlo simulations

The simulations of the charge transport process in a thin film consisting of the MD-constructed amorphous model of CBP were carried out by kMC method implemented in our in-house program. All simulations were performed at 300 K with a periodic boundary condition until carriers migrated a distance (*L*) of 100 nm along the applied electric field ***F*** (|***F***| = *F*). ***F*** was set in the same direction as the *x*-, *y*-, or *z*-axis. The calculated mobilities for the three orthogonal directions were essentially the same, reflecting the isotropic nature of the amorphous system. The calculated mobilities provided in this study are the average values for these directions. For each kMC trials, we inject one carrier on a randomly selected molecule in the amorphous aggregate and simulated the trajectory under the applied electric field of ***F***. The inter-carrier electrostatic interaction was ignored in the simulations. According to refs^46–49^, charge mobilities do not depend on carrier densities when they are lower than 10^15^–10^16^ cm^−3^. This condition meets both of our kMC simulations and TOF experiments. The situation is significantly different from the higher charge density regime (> 10^17^ cm^−3^) where the mobility obviously increases with increasing charge density and electrostatic interactions play an important role. We simulated 10^4^ trajectories of the carrier per direction for each of the *x*-, *y*-, and *z*-directions (3 × 10^4^ trajectories in total) to determine the transit time *t*. The mobility *μ* was calculated from *μ* = *L */ (*tF*). Supplementary Fig. S2 shows a comparison of the calculated mobilities for CBP-4000-A and CBP-4000-B.

## Electronic supplementary material

Supplementary Information

## Data Availability

The datasets generated during and/or analysed during the current study, including the amorphous models, computed charge transport parameters, statistical results of kMC simulations, are available from the corresponding author on reasonable request.
