# Supplementary material for: Parameter-Free Multiscale Simulation Realising Quantitative Prediction of Hole and Electron Mobilities in Organic Amorphous System with Multiple Frontier Orbitals
Source: Sci Rep. 2018 Sep 7;8:13462. doi: 10.1038/s41598-018-31722-w (PMC6128853; doi:10.1038/s41598-018-31722-w)
Supplement: Supplementary file 1 — Supplementary Information [file 41598_2018_31722_MOESM1_ESM.pdf]

Supplementary Information for: Parameter-Free Multiscale Simulation Realising Quantitative Prediction of Hole and Electron Mobilities in Organic Amorphous System with Multiple Frontier Orbitals

Shosei Kubo and Hironori Kaji*

Institute for Chemical Research, Kyoto University, Uji, Kyoto 611-0011, Japan

E-mail: kaji@scl.kyoto-u.ac.jp

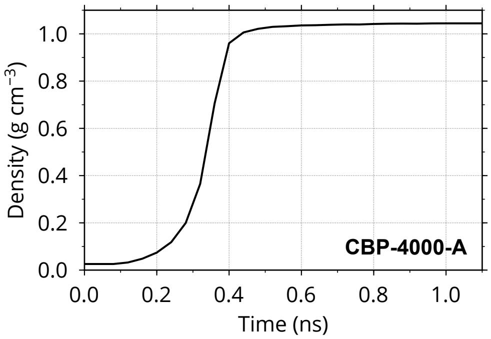

Supplementary Figure S1: Change in the density of the amorphous aggregate consisting of 4,000 CBP molecules, CBP-4000-A, during the MD simulation. A similar result was obtained for CBP-4000-B.

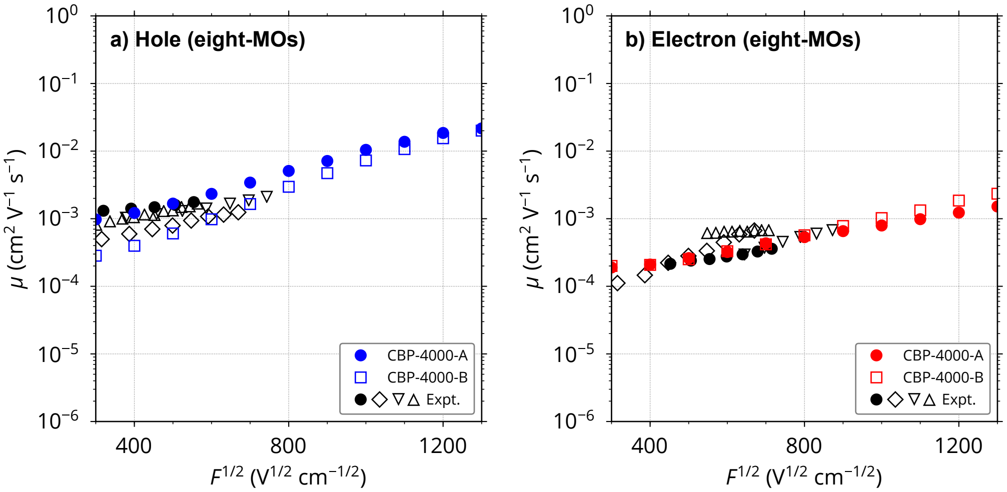

Supplementary Figure S2: Charge mobility μ calculated for the two MD-constructed amorphous models of CBP, CBP-4000-A and CBP-4000-B, for (a) hole and (b) electron transport in the eight-MO model. The experimental data are shown as black symbols with the same notation as in Fig. 4 in the main text. Although slight variation of the calculated hole mobilities was found depending on the final structure of the amorphous model, the calculated mobilities were almost within the variation obtained experimentally.

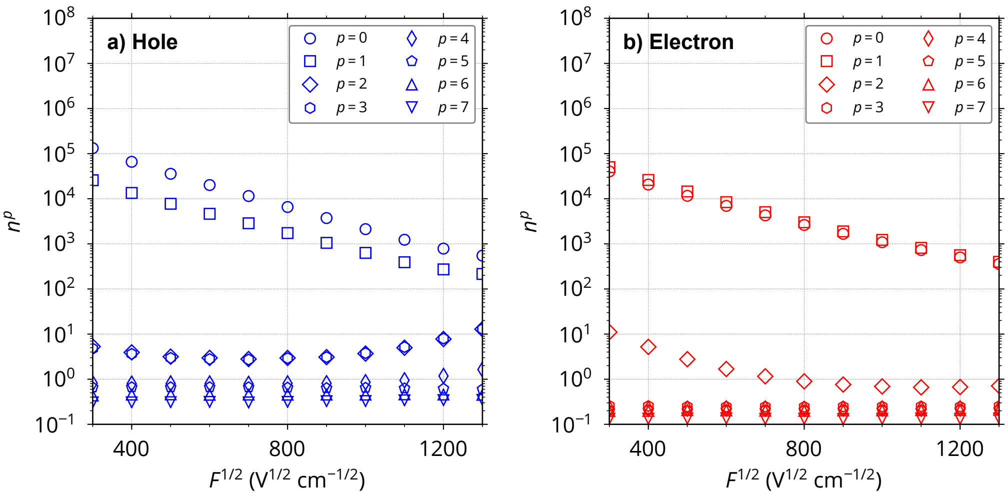

Supplementary Figure S3: Number of incoming carriers from all adjacent MOs to pth MOs in the amorphous system, n^p, as a function of $F^{1/2}$ for (a) hole and (b) electron transport in the eight-MO model. n^p is averaged over 30,000 trials of the kMC simulation.

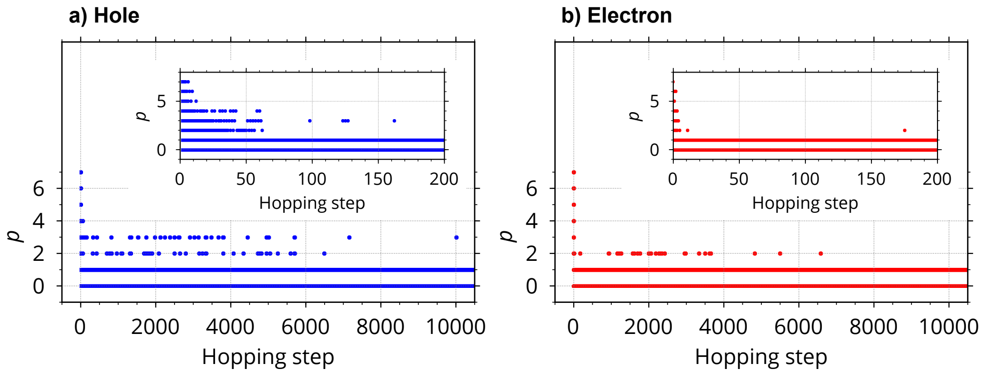

Supplementary Figure S4: Change in charge occupation of pth MOs for (a) hole and (b) electron transport in the eight-MO model. 100 trials of the kMC simulation at $F^{1/2}$ = 800 V$^{1/2}$ cm$^{-1/2}$ are superposed. The first 200 hopping steps are expanded in the inset.

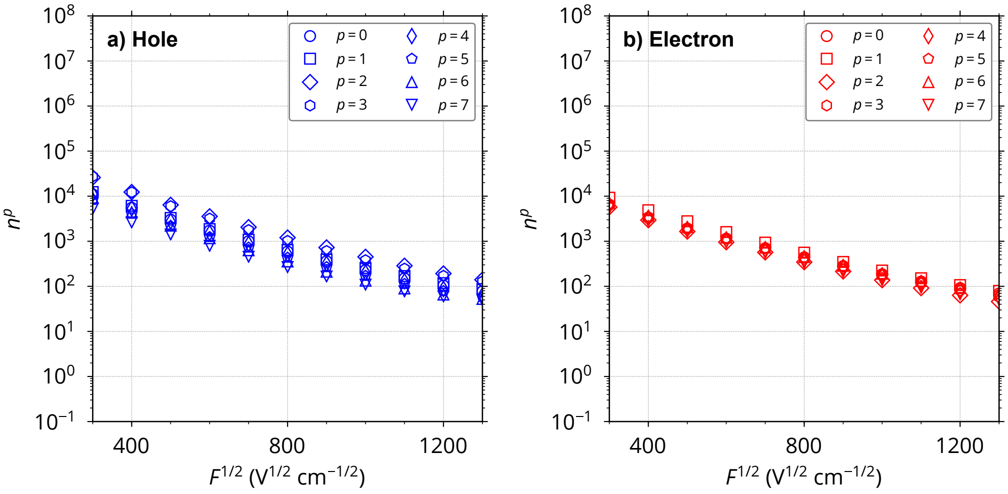

Supplementary Figure S5: Number of incoming carriers from all adjacent MOs to pth MOs in the amorphous system, n^p, as a function of $F^{1/2}$ for (a) hole and (b) electron transport where all eight MOs, HOMO to HOMO−7 or LUMO to LUMO+7, are quasi-degenerate. n^p is averaged over 30,000 trials of the kMC simulation.

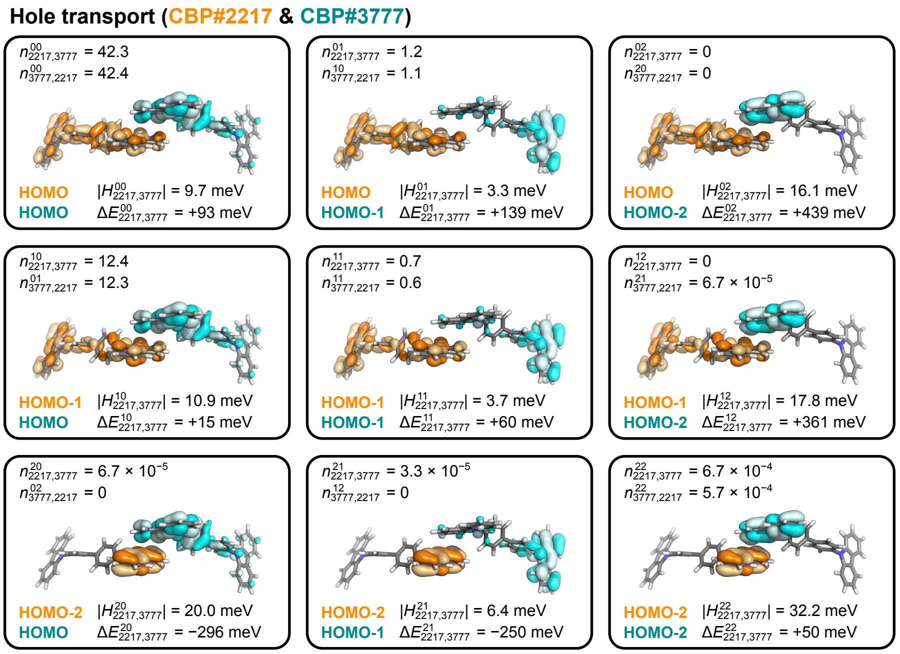

Supplementary Figure S6: Examples of MO pairs for hole transport with $p \leq 2$ (HOMO to HOMO−2), depicted with an isosurface of 0.02 a.u. The electronic coupling $|H^{pq}_{ij}|$, site energy difference ΔE^{pq}_{ij}, and number of hops between the pth MO of the ith molecule and the qth MO of the jth molecule, n^{pq}_{ij} and n^{qp}_{ji}, for each pair are also provided. n^{pq}_{ij} and n^{qp}_{ji} are averaged over 30,000 trials of the kMC simulation at $F^{1/2} = 800\ V^{1/2}\ cm^{-1/2}$.
